# Nonlinear Conductive Characteristics of ZnO-Coated Graphene Nanoplatelets-Carbon Nanotubes/Epoxy Resin Composites

**DOI:** 10.3390/polym12081634

**Published:** 2020-07-23

**Authors:** Yang Yuan, Zhaoming Qu, Qingguo Wang, Xiaoning Sun

**Affiliations:** National Key Laboratory on Electromagnetic Environment Effects, Army Engineering University, Shijiazhuang 050003, China; spirit_yugi@163.com (Y.Y.); iamqzm3990@163.com (Z.Q.); sunxiaoning_8@163.com (X.S.)

**Keywords:** nonlinear conductive characteristics, polymeric composites, switching threshold voltage, reversibility, GNPs-CNTs-ZnO hybrid

## Abstract

With the increasing threats arising from the electromagnetic environment, polymeric composites which could exhibit nonlinear conductive characteristics are highly required in the protection of electronic devices against overvoltage. In this research, ZnO nanoparticles are coated onto graphene nanoplatelets (GNPs)-carbon nanotubes (CNTs) hybrid, and then it is embedded in epoxy resin (ER) matrix via solution blending. Based on the characterization results, CNTs are well dispersed across the GNPs which prevent the restacking of GNPs and CNTs. At the same time, ZnO nanoparticles are well-bonded to the surfaces of GNPs-CNTs hybrid. During repeated conductive characteristic measurements, GNPs-CNTs-ZnO/ER composite is able to demonstrate distinctly reversible nonlinear conductive behavior, with high nonlinear coefficients. Especially, the filler content in GNPs-CNTs-ZnO/ER composite is only 12.5% of that in GNPs-ZnO/ER composite reported in our previous work. Moreover, it is shown that the nonlinear coefficients and switching threshold voltage can be modified by controlling the weight ratios of GNPs, CNTs, and ZnO. Finally, the samples with 1:1 weight ratio of GO to MWCNTs (A-6.67 and A-10) exhibit the best reversible nonlinear conductive behavior.

## 1. Introduction

Materials with distinct and stable nonlinear conductive characteristics are a class of materials that could be operated as an insulator under normal conditions, and could be converted to a conductor when the applied voltage reach the critical threshold [1,2,3]. Such materials have been used as efficient methods to safeguard electronic devices from the damages of voltage surge and electrostatic discharge. Thus, these materials have attracted tremendous interest in recent years as a potential way to effectively enhance the reliability and safety of electronic devices [4].

As shown in previous reports, polymeric composites, comprising of highly stable polymer matrix and fillers with good electrical conductivity via a series of proper reaction methods, are successful to demonstrate obvious nonlinear conductive behaviors [5,6,7,8,9,10], which indicates the potential applying of polymeric composites for preventing the overvoltage of electronic devices [11,12,13]. For instance, White et al. [14,15] demonstrated the preparation and characterization analysis of silver nanowire-polystyrene filler nanocomposites. In addition, they further analyzed the reversible voltage-induced conductive switching behavior of the composite at room temperature.

Because of their flexibility and superior properties, both graphene nanoplatelets (GNPs) and carbon nanotubes (CNTs) are widely used as efficient enhancing fillers [16,17]. Between the two materials, GNPs have drawn extensive research interest as a promising conductive filler due to their unique 2D-layer microstructure and superb electrical conductivity of 6 × 10^5^ S/m [18]. On one hand, recent studies have indicated that the conductivity and dispersibility of few-layer graphene could be enhanced with modified methods, so the modified graphene could efficiently improve the electrical properties of the resulting polymer composite [19,20,21,22,23,24]. On the other hand, CNTs, including single walled carbon nanotubes (SWCNTs) and multiwalled carbon nanotubes (MWCNTs), are prized for their special 1D-linear microstructure and distinct mechanical, chemical, and electrical properties. Similar to GNPs, CNTs are also considered as a promising filler in the fabrication of high-performing polymeric composite [25,26,27,28,29].

Despite the multiple advantages of GNPs and CNTs, they both tend to restack during the preparation process due to their exceedingly large surface area, and this restacking process could severely undermine the excellent characteristics of composites. However, according to the recent reports, such a challenge can be mitigated with proper processing method.

ZnO is a popular wide-gap semiconductor, which possesses various excellent characteristics in field-induced phase transition, in particular for its reversibility. For instance, it was demonstrated by Yu et al. that a polymer containing ZnO-decorated CNT composites could exhibit a reversible nonlinear I–V behavior [30]. Motivated by this encouraging result, it is expected that ZnO can effectively enhance the reversibility of GNPs-filled and CNTs-filled composite by coating on GNPs-CNTs hybrids.

Herein, in this work, ZnO nanoparticles coated GNPs-CNTs hybrids are prepared, via a one-step solvothermal method, as the filler to fabricate the composite with nonlinear conductive characteristics. According to the morphological analysis and conductive characteristic measurements, GNPs-CNTs-ZnO hybrids and their composites exhibit good microstructure and distinct nonlinear conductive behavior under a particular applied voltage. The as-obtained sample is not only able to demonstrate a far lower threshold voltage as compared to the ZnO-based ceramic varistors, it is also able to exhibit excellent reversibility in its nonlinear conductive behavior after multiple measurements. Interestingly, it is shown that the threshold voltage of GNPs-CNTs-ZnO/epoxy resin (ER) composite can be controlled by adjusting the weight ratios of graphene oxide (GO) to MWCNTs and GNPs-CNTs hybrid to Zn(Ac)_2_. This observation means that the reversible nonlinear conductive behavior of the composite can be modulated with a few parameters. Furthermore, when comparing with our previous work on GNPs-ZnO/ER composite [31], the required weight ratio of the fillers in GNPs-CNTs-ZnO/ER composite is 12.5% that of GNPs-ZnO/ER composite [31,32]. This lower amount of filler required in this article indicates that the cost of composite fabrication can be effectively decreased. Finally, the conductive mechanism of the nonlinear conductive behavior of the GNPs-CNTs-ZnO/ER composite is elucidated in this work. It is shown that GNPs-CNTs-ZnO/ER composite is tailored for the practical protection against overvoltage of electronic devices due to their stably reversible nonlinear conductive characteristics and low fabrication cost. This could assure the standard operation of the electronic devices, and effectively decreasing the risk of damaging the device caused by repeated voltage surges.

The novelty of this work is the employment of three components with different phases as the filler in the GNPs-CNTs-ZnO/ER composite, which has not yet been reported in this field. This is in stark contrast to the previous reports whereby most GNPs composites contain either one or two type of fillers. In addition, the GNPs-CNTs-ZnO/ER composite reported in this work requires lower fabrication cost and it is able to demonstrate excellent reversibility and stability as compared to other nonlinear conductive composites. Furthermore, there are more available methods to control the reversible nonlinear conductive characteristics of GNPs-CNTs-ZnO/ER composite, which makes it highly tailorable. Thus, this work is expected to carve a novel and more practical approach in the enhancement of the durability and safety of the electronic equipment.

## 2. Materials and Methods

### 2.1. Materials

Graphene oxide, used as the precursor to GNPs, and multiwalled carbon nanotubes (with external diameter of 30–50 nm and length of 10–20 μm), used as the precursor to CNTs, were obtained from Tanfeng Tech Company (Suzhou, China). Zinc acetate (Zn(Ac)_2_∙2H_2_O), used as the precursor to ZnO, and ethyl alcohol, used as the main solvent, were obtained from Yongda Chemical Reagent Company (Tianjin, China). Epoxy resin (E-51), used as insulating polymeric matrix, was obtained from Hui-Sheng Electronic Material Company (Chuzhou, China). The 2-ethyl-4-methylimidazole (2E4MZ) (analytical reagent, purity: 99%), employed as the curing agent of E-51, was purchased from Xiya Reagent Company (Chengdu, China). Sodium hydroxide (NaOH), employed to adjust the pH of the reaction system, was purchased from Dalu Chemical Reagent Company (Tianjin, China). Hydrazine hydrate solution (analytical reagent, mass fraction: 85%), used as the reducing agent, was obtained from Sinopharm Chemical Reagent Company (Tianjin, China).

### 2.2. Materials Preparation

To improve the dispersity of MWCNTs, a particular amount of MWCNTs was added into the mixture of nitric acid and sulfuric acid (3:1 in volume), and the system was stirred for four hours at 65 °C. After which, the CNT/acid mixture was subjected to an ice water bath, and it was neutralized with NaOH later. After the suspension was leached for three times, the obtained filter cake was freeze dried for 24 h to acquire the purified CNTs.

To disperse GO-MWCNT well in Zn(CH_3_COO)_2_·2H_2_O solution, GO powders and purified CNTs were firstly added into ethyl alcohol and the mixture was ultrasonicated for 1 h. Next, Zn(CH_3_COO)_2_·2H_2_O was then added into the GO-CNTs solution, and the mixture was subjected to another hour of ultrasonication. The pH of the system was then adjusted to 10 by adding NaOH dropwise. Then, the system was magnetically stirred for 1 h. After stirring for 1 h, a small amount of hydrazine hydrate was introduced into the system, and it was stirred for 6 h at 90 °C to obtain the GNPs-CNTs-Zn(OH)_2_ suspension. The mixture was then placed in a Teflon-lined stainless steel autoclave and undergoes solvothermal reaction for 20 h at 180 °C. After the completion of the solvothermal reaction, the reaction system was cooled down and leached for three times. Finally, the filter cake was freeze dried for 24 h to acquire the GNPs-CNTs-ZnO hybrid.

Solution blending with acetone was used to prepare the GNPs-CNTs-ZnO/ER composite. The mixture was stirred for several hours at 80 °C, until the acetone was fully evaporated. Then, a small amount of 2E4MZ was introduced into the system as a curing agent. After stirring and vacuuming, the reaction system was transferred into a disposable dish so as to allow it to be cured for certain duration and temperature, i.e., 24 h at 20°C and 4 h at 100 °C, to finally acquire the GNPs-CNTs-ZnO/ER composite.

### 2.3. Characterization and Measurements

The microstructural and morphological analysis of GNPs-CNTs-ZnO hybrid and the fracture surface of GNPs-CNTs-ZnO/ER composite were performed using a transmission electron microscope (TEM, JEOL JEM-2100, Tokyo, Japan) and scanning electron microscope (SEM, GeminiSEM 300, Jena, Germany). X-ray diffraction (XRD) of the GNPs-CNTs-ZnO hybrid was determined using PuXi XD-6 (Beijing, China). Fourier transform infrared spectrums (FTIR) of GNPs and GNPs-CNTs-ZnO hybrid were recorded using GangDong FTIR-650 (Tianjin, China) to analyze their oxygen functionalities and their reduction degrees. Raman spectrum of the GNPs-CNTs-ZnO hybrid was recorded using a Raman spectrometer (Horiba Scientific LabRAM HR Evolution, Tokyo, Japan). The atomic composition of the GNPs-CNTs-ZnO hybrid, i.e., carbon, oxygen, and zinc, was investigated by an X-ray photoelectron spectrometer (XPS, Thermo Scientific K-Alpha+, Waltham, MA, USA).

A Keithley 2600-PCT-4B semiconductor parameter analyzer (Cleveland, OH, USA) was employed to record the conductive characteristics of GNPs-CNTs-ZnO/ER composite. To ensure a good contact between the sample and test tool, both surfaces of the sample were coated with a thin layer of silver conductive resin.

## 3. Results

### 3.1. Characterization of GNPs-CNTs-ZnO Hybrid and Its Composite

**Figure 1:**
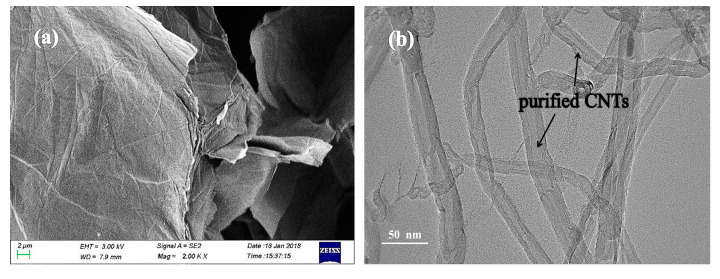
SEM image (**a**) of graphene oxide (GO) powder and TEM image (**b**) of purified carbon nanotubes (CNTs).

The SEM image of the GO powder and the TEM image of purified CNTs, i.e., the precursors to GNPs and CNTs, respectively, are shown in Figure 1. Based on Figure 1a, most of GO flakes possess single layer structure without visible defects or re-stacking. The TEM image of purified CNTs (Figure 1b) reveals that the as-prepared purified CNTs possess good microstructure with a rather uniform external diameter and flat surface. Thus, based on the results of SEM and TEM, the GO powder and purified CNTs are highly suitable as the precursors to the fabrication of GNPs and CNTs, and could satisfy the need of ZnO decoration, which lays the foundation for the subsequent processes.

**Figure 2:**
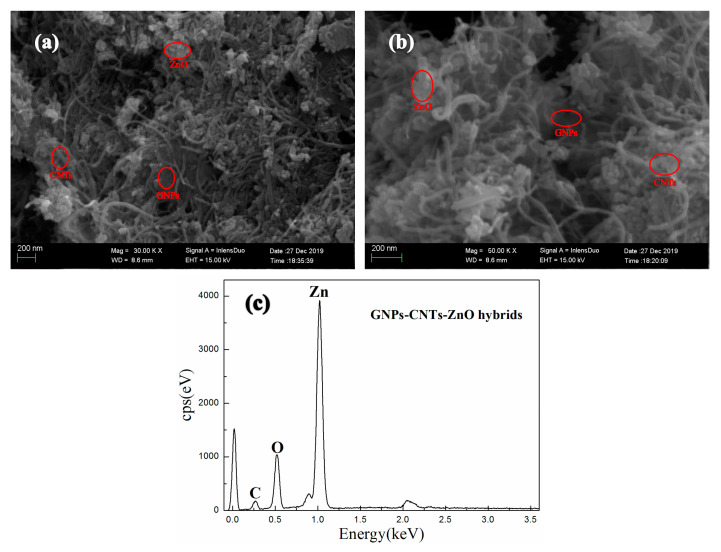
SEM images (**a**,**b**) and EDS (**c**) of graphene nanoplatelets (GNPs)-CNTs-ZnO hybrids.

The SEM images and energy dispersive spectroscopy (EDS) analysis of GNPs-CNTs-ZnO hybrid are presented in Figure 2. There is almost no re-stacking in hybrids can be observed in Figure 2a, while CNTs and ZnO nanoparticles are evenly dispersed across the GNPs. This may be due to the presence of Van der Waals interaction between the GNPs and CNTs, which can prevent the re-stacking of GNPs and CNTs. Furthermore, because of the electrostatic attraction, Zn^2+^ are adhered to the oxygen functionalities on the surface of GO and purified CNTs. This could help to retain a good surface morphology during the reduction process. On the other hand, GNPs-CNTs-ZnO hybrids are reduced into a smaller size during the reaction as compared to GO, and the majority of those exist as few-layer structure with good morphological characterization. Furthermore, according to the high magnification SEM image, presented in Figure 2b, it is shown that more ZnO nanoparticles are decorated on the GNPs as compared to that on the CNTs. This may be due to the larger exposed surface area and the greater content of oxygen-containing groups on the surfaces of GNPs as compared to CNTs. However, as GNPs play a more important role in terms of conductivity, it can be considered that ZnO nanoparticles are successfully and evenly coated on the GNPs-CNTs hybrids.

EDS is conducted for GNPs-CNTs-ZnO hybrids, and the result is shown in Figure 2c. According to the EDS analysis, the main elements of the sample are Zn, C, and O, which hints that ZnO nanoparticles are successfully obtained and decorated on the GNPs-CNTs hybrids.

**Figure 3:**
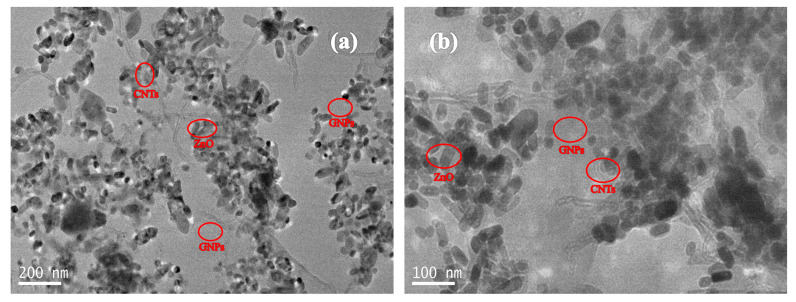
TEM image (**a**) and high magnification TEM image (**b**) of the GNPs-CNTs-ZnO hybrid.

The TEM images of GNPs-CNTs-ZnO hybrid are presented in Figure 3. Based on Figure 3a, GNPs are able to retain high specific surface area with a low amount of defects after the preparation process, i.e., reduction and solvothermal reaction, because of the protection of the CNTs and Zn^2+^. When compared with Figure 1, it can be observed that all CNTs and ZnO nanoparticles are successfully decorated on the surface of GNPs. Furthermore, the bonding points of ZnO nanoparticles are the oxygen-containing groups on the surfaces of GO and purified CNTs. These are mainly located at the edge of GNPs, and a larger specific surface area could translate to a greater likelihood of being bonded with ZnO nanoparticles. According to the high magnification TEM image presented in Figure 3b, ZnO nanoparticles are confined on the GNPs, while lesser amount of these ZnO nanoparticles are present on the surface of CNTs. This observation is consistent with the earlier mentioned hypothesis. However, as ZnO nanoparticle coating is to avoid the direct contact of the closely distributed GNPs and CNTs, ZnO nanoparticles with size of 50–60 nm are well bonded to the surface of the GNPs and CNTs, as shown in Figure 3.

**Figure 4:**
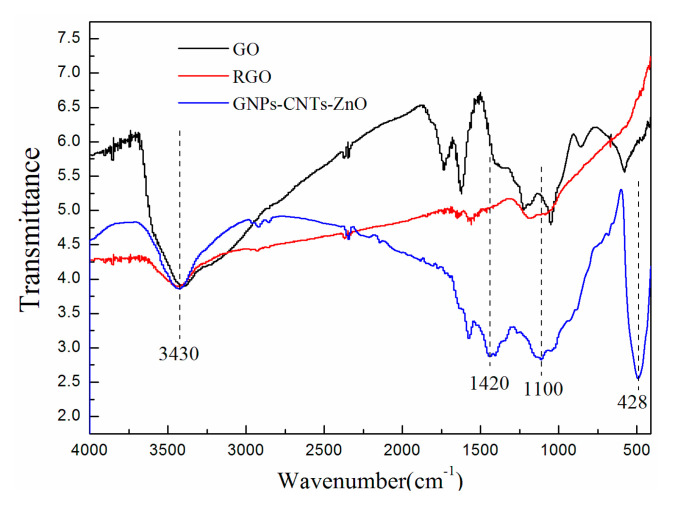
FTIR spectrums of GO, reduced graphene oxide (RGO), and GNPs-CNTs-ZnO hybrid.

The oxygen functionalities on GO and purified CNTs can be removed by reducing agent for enhancing the conductivity of them. During the reduction of GO to GNPs and purified CNTs to CNTs, sp^3^-bonded structures are transformed to sp^2^-bonded structures, which can significantly enhance the conductivity of the GNPs-CNTs hybrid. Simultaneously, this reduction process will inevitably result in the generation of more defects on the surface of GNPs and CNTs [33,34] When comparing Figure 2a with Figure 3, it can be observed that high temperature and use of reducing agent during the reduction and synthesis of GNPs−CNTs-ZnO hybrid, lead to the more obvious re-stacking and formation of wrinkles in the GNPs-CNTs hybrid. FTIR is used to investigate the reduction process of GNPs-CNTs-ZnO hybrid by recording the FTIR spectrums of GO, reduced graphene oxide (RGO), and GNPs-CNTs-ZnO. Based on Figure 4, the peaks of RGO and GNPs-CNTs-ZnO are weaker than those of GO. On the other hand, for GNPs-CNTs-ZnO, the peaks located at 1100 cm^−1^ (C-O-C stretching vibration), 1420 cm^−1^ (C=O stretching vibration), and 3430 cm^−1^ (O-H stretching vibration) are stronger as compared to those of RGO. This result suggests that a greater content of oxygen functionalities are present in GNPs-CNTs-ZnO hybrid as compared to RGO. This is because the bonding of ZnO to GNPs-CNTs hybrids can prevent some oxygen functionalities from being eliminated. Interesting, a distinct Zn-O stretching vibration at 428 cm^−1^ can be observed, which further verifies the successful synthesis of ZnO nanoparticles on the surface of GNPs-CNTs hybrids.

**Figure 5:**
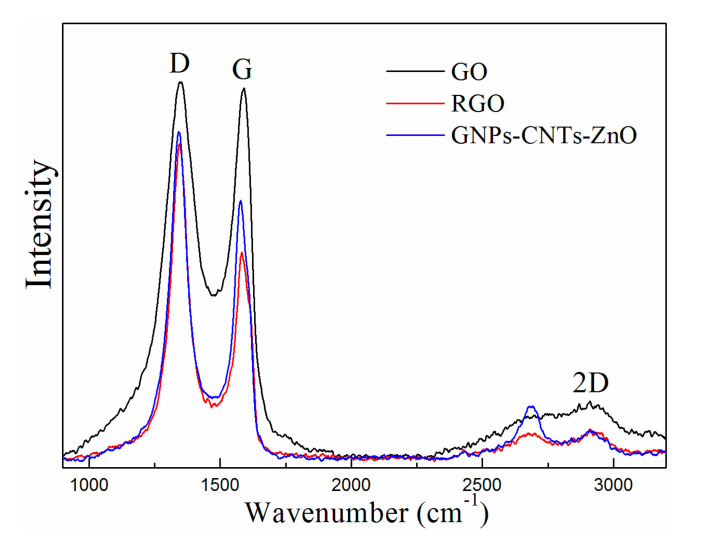
Raman spectrums of GO, RGO, and GNPs-CNTs-ZnO hybrid.

The Raman spectrums of GO, RGO, and GNPs-CNTs-ZnO hybrid, and their respective D-band (disorder band, 1341.96 cm^−1^) and the G-band (sp^2^ carbon, at 1578.06 cm^−1^) are shown in Figure 5. D-band is the resultant of the defects in the structure of materials, e.g., sp^3^ bonds, presence of small sized crystalline domains, and functional groups [35,36] The estimation of the I_D_/I_G_ values can give some insight to the disorder degree of the material. The calculated I_D_/I_G_ values of GO, RGO, and GNPs-CNTs-ZnO hybrid are 1.017, 1.518, and 1.266, respectively. This result indicates that as the oxygen functionalities on GO and purified CNTs are removed during the reduction process, more defects, e.g., holes on the surfaces of GNPs and CNTs, are generated in the samples, which leads to the higher disorder degrees in RGO and GNPs-CNTs-ZnO hybrid. Moreover, it can be observed that the I_D_/I_G_ value of GNPs-CNTs-ZnO hybrid is smaller than that of RGO. This may be due to the ZnO nanoparticle coating which could help to reduce the extent of defect generated in the material. This result further indicates the successful bonding between ZnO nanoparticles with the surface of GNPs and CNTs.

**Figure 6:**
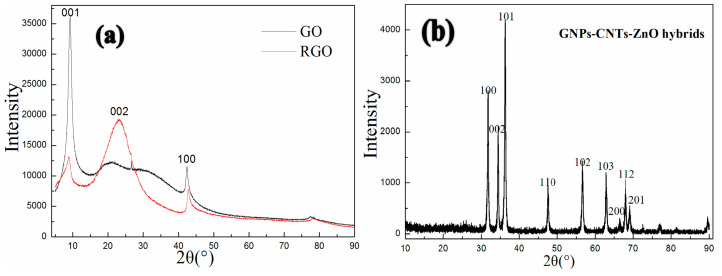
XRD spectrums of GO, RGO (**a**) and GNPs-CNTs-ZnO hybrid (**b**).

The XRD spectrums of GO, RGO, and GNPs-CNTs-ZnO hybrid are displayed in Figure 6. From Fig6a, the peaks located at 10.6° and 42.8° are indexed to the characteristic peaks, which respectively correspond to the (001) and (100) crystal planes of GO and indicate the well crystal structure of GO. Moreover, according to the XRD spectrum of RGO, the new peak located at 24.8°, indexed to the (002) crystal planes of well crystalline graphene, and weaker peaks located at 10.6° and 42.8°, indicate GO has been successfully reduced to RGO, which is in accord with the result of FTIR. Meanwhile, based on the XRD spectrum Figure 6b, all the peaks located at 31.5°, 34.1°, 36.3°, 47.1°, 56.4°, 62.6°, 66.5°, 67.6°, and 69.1° can be well-indexed to the characteristic peaks of hexagonal ZnO structure (JCPDS No. 36–1451), which correspond to the (100), (002), (101), (110), (102), (103), (200), (112), and (201) crystal planes of ZnO, respectively. Compared with Figure 6a, no impurity peaks can be observed in Figure 6b, which indicates the successful formation of hexagonal ZnO (wurtzite) nanoparticles that are well coated onto the surface of GNPs-CNTs hybrids.

**Figure 7:**
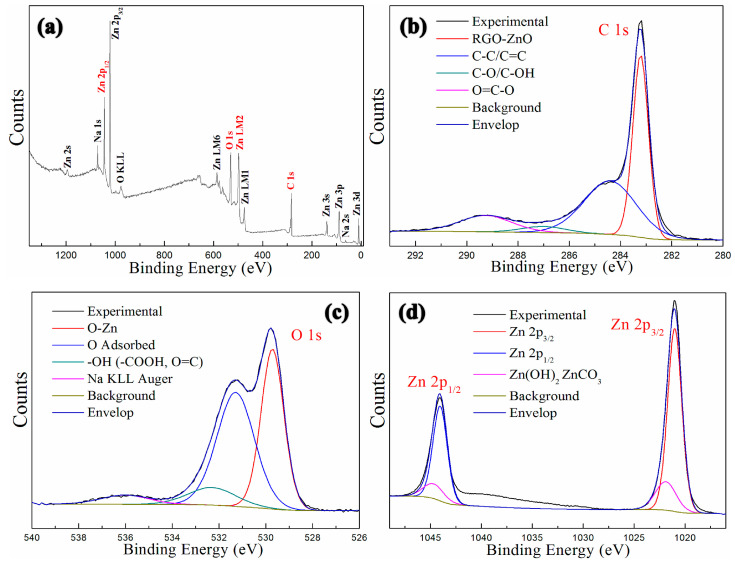
XPS spectrums of GNPs-CNTs-ZnO hybrid: Survey spectrum (**a**), C 1s (**b**), O 1s (**c**), and Zn 2p (**d**).

The XPS spectrums of GNPs-CNTs-ZnO hybrid are shown in Figure 7. Figure 7a presents the survey XPS spectrum of GNPs-CNTs-ZnO hybrid, whereby several binding energy peaks, e.g., carbon (C 1s, 296.8 eV), zinc (Zn 2p_3/2_, 1028.1 eV, and Zn 2p_1/2_, 1040.2 eV), and oxygen (O 1s, at 532.6 eV). The high resolution C 1s XPS spectrum of GNPs-CNTs-ZnO hybrid, shown in Figure 7b, is in the range of 281–292 eV. It can be observed that the peak area of C-C/C=C (284.4 eV) is much larger as compared to those of C-O/C-OH (287.1 eV) and O=C-O (289.3 eV). This result suggests that most oxygen functionalities on GO and purified CNTs are eliminated during the synthesis of GNPs-CNTs-ZnO hybrid. In particular, a distinct peak at 283.2 eV can be observed for RGO-ZnO which can be attributed to the bonding between GNPs-CNTs hybrids and ZnO. This result further verifies the successful formation of ZnO nanoparticles on GNPs-CNTs hybrid with strong bonds.

The high-resolution O 1s XPS spectrum of GNPs-CNTs-ZnO hybrid is displayed in Figure 7c, with a binding energy range of 526–540 eV. Based on this result, the peak at 529.6 eV is distinct which is indicative of Zn-O. The high-resolution Zn 2p XPS spectrum of GNPs-CNTs-ZnO hybrid is shown in Figure 7d, with a binding energy range of 1015–1050 eV. The high-resolution Zn 2p XPS spectrum includes Zn 2p_3/2_ and Zn 2p_1/2_ at 1020.0 eV and 1044.1 eV, respectively. Based on the combined results from Figure 7c,d, it can be deduced that well-crystallized ZnO nanoparticles are formed on the surface of GNPs-CNTs hybrid.

**Figure 8:**
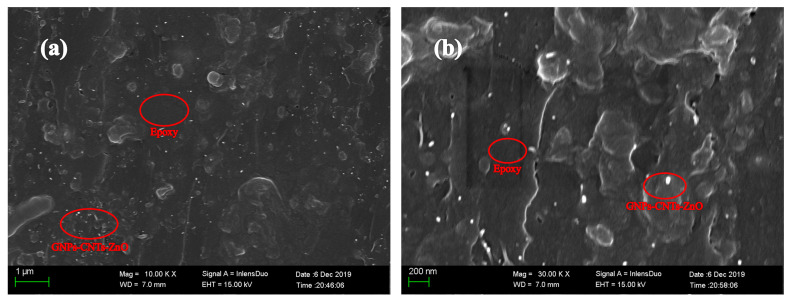
SEM image (**a**) and high magnification SEM (**b**) of the fracture surface of GNPs-CNTs-ZnO/epoxy resin (ER) composite.

The fracture surface of GNPs-CNTs-ZnO/ER composite is observed under SEM at various magnifications, and the results are shown in Figure 8. It can be observed that GNPs-CNTs-ZnO hybrids are dispersed evenly through the ER matrix. Furthermore, agglomeration and the surface between GNPs-CNTs-ZnO hybrid and ER are negligible, which suggest the excellent compatibility and dispersity of GNPs-CNTs-ZnO hybrid.

### 3.2. Reversible Nonlinear Conductive Behavior of GNPs-CNTs-ZnO/ER Composite

To investigate the conductive behavior of the as-obtained GNPs-CNTs-ZnO/ER composite, all tested samples are categorized into three groups (A, B, C) based on the weight ratio of GO to MWCNTs, i.e., 1:1, 1:2, and 2:1. Within each group, it is further categorized into four individual samples based on the weight ratio of GNPs-CNTs hybrid to Zn(Ac)_2_, i.e., 1:20, 1:10, 1:6.67, and 1:5. Then, several specimens with the GNPs-CNTs-ZnO hybrids filler concentration of 2.5% are obtained. The interval between two consecutive conductive characteristic measurements is set as 30 s. Especially, the classification and allographs of all samples are shown in Table 1.

**Figure 9:**
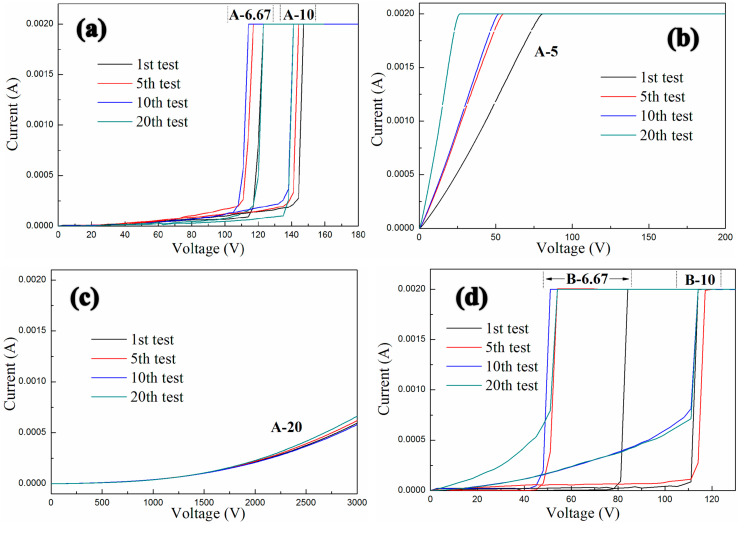

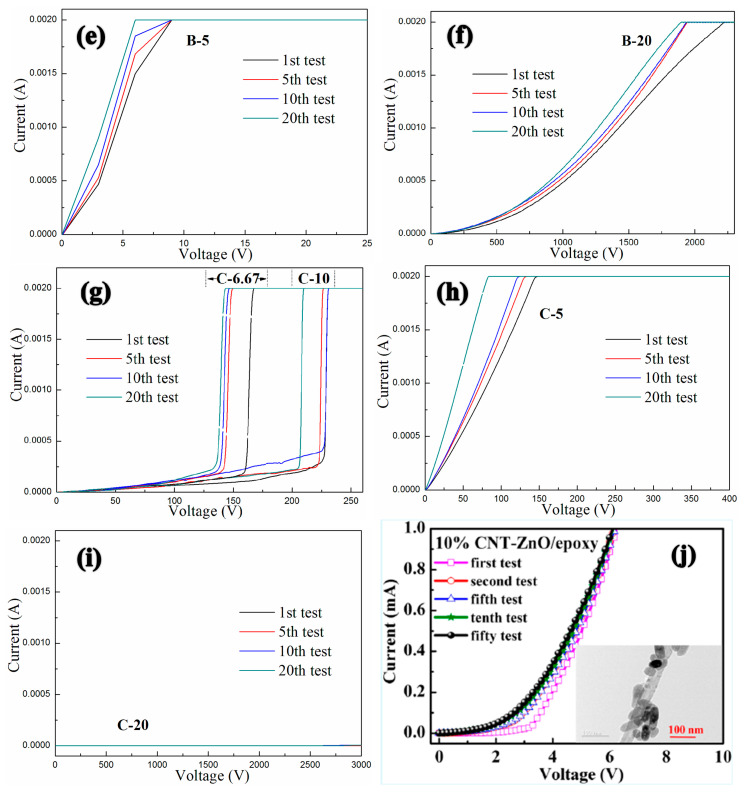
Conductive characteristics of the three groups of GNPs-CNTs-ZnO/ER samples: (**a–c**) with 1:1 weight ratio of GO to MWCNTs, (**d–f**) with 1:2 weight ratio of GO to MWCNTs, (**g–i**) with 2:1 weight ratio of GO to MWCNTs, and (**j**) the composite fabricated by Yu et al.

The conductive behaviors of GNPs-CNTs-ZnO/ER composites with different weight ratios of GO to MWCNT and GNPs-CNTs hybrid to Zn(Ac)_2_ is shown in Figure 9. As shown in Figure 9a, two different samples A-6.67 and A-10 both exhibit linear ohmic behavior at low voltage (Region 1) and an obvious non-ohmic behavior (Region 2) at relatively high voltage. With the increase in the weight ratio of GNPs-CNTs hybrid to Zn(Ac)_2_, the boundary of Region 1 and Region 2, i.e., the switching threshold voltage, obviously decreases. Furthermore, in contrast to the one-off conductive behavior in traditional GNPs composite, the two GNPs-CNTs-ZnO/ER samples (A-6.67 and A-10) shown in Figure 9a not only exhibit distinct nonlinear conductive behaviors at high applied voltages, they are also able to demonstrate stable reversibility throughout all 20 measurements. In particular, due to the first transformation of small number of thin ER matrix between ZnO nanoparticles from insulator to conductor under applied voltage, there are some slight deviations in the initial measurements of the two samples. As shown in Figure 9d,g, four other different GNPs-CNTs-ZnO/ER samples (B-6.67, B-10, C-6.67, and C-10), all exhibit similar reversible nonlinear conductive behavior throughout 20 measurements. Meanwhile, an obvious increase in the switching threshold voltage of the sample with same weight ratio of GNPs-CNTs hybrid to Zn(Ac)_2_ can be observed with the increase in the weight ratio of GO to MWCNTs.

In addition, Figure 9b,c show two other cases. Figure 9b shows the conductive behavior of the GNPs-CNTs-ZnO/ER sample (A-5). It is revealed that this sample only exhibits linear ohmic behavior throughout 20 measurements, with increasing slope in the curves. This is because the content of filler in GNPs-CNTs hybrid is highly excessive which cannot be effectively coated by ZnO nanoparticles, hence resulting in poor reversibility that is exhibited by pristine GNPs/ER composite. By comparing Figure 9e,h, the slopes of the curves for two samples (B-5 and C-5) are proportional to the filler concentration in the GNPs-CNTs hybrid.

On the contrary, as shown in Figure 9c, the sample (A-20) can only exhibit a slight trend of nonlinear conductive behavior, and it is not able to transform as conductor under extremely high applied voltage (3000 V). This observation may be attributed to the insufficient filler concentration of the GNPs-CNTs hybrid. When compared to Figure 9c, as shown in Figure 9f, the sample (B-20) exhibits higher conductivity and unobvious nonlinear conductive behavior as well. Meanwhile, the sample (C-20), as shown in Figure 9i, exhibits almost no transformation throughout the 20 measurements.

The nonlinear conductive coefficient, *α,* of the material can be estimated by calculating the ratio of log(*I*_2_/*I*_1_) to log(*V*_2_/*V*_1_) according to the following equation, *α* = [log(*I*_2_) − log*(I*_1_)]/[log(*V*_2_) − log(*V*_1_)], where *I*_1_ is the measured current at *V*_1_, and *I*_2_ is the measured current at *V*_2_ [30].

The average *α* of different GNPs-CNTs-ZnO/ER samples with various weight ratios of GNPs to CNTs and GNPs-CNTs hybrid to Zn(Ac)_2_ after several measurements is shown in Table 2. It can be observed that, for all six samples, *α* of Region 2 are larger as compared to that of Region 1. This indicates that all the as-prepared GNPs-CNTs-ZnO/ER samples exhibit very distinct nonlinear conductive behavior. Furthermore, based on the six average *α* of the Region 2 that are shown in Table 2, an increase in the weight ratio of GO to MWCNTs can lead to a significant increase in *α*. This trend is due to the initially lower conductivity of the samples, which leads to the highest *α* of Region 2 for the group with 2:1 weight ratio of GO to MWCNTs (Group C).

Table 3 presents the range of the change in the switching threshold voltage and the standard deviation Δ of six different GNPs-CNTs-ZnO/ER samples. As shown in Table 3, as the weight ratio of GO to MWCNTs decreases, the switching threshold voltage decreases as well. This trend could be due to the initially higher conductivity of the samples, whereby the group with the 1:2 weight ratio of GO to MWCNTs (Group B) exhibits the smallest switching threshold voltage. Meanwhile, the group with the 2:1 weight ratio of GO to MWCNTs (Group C) possesses the most stable reversibility of nonlinear conductive behavior after repeated measurements, as it has the smallest average standard deviation value.

According to relevant research of Yu et al., the polymeric composites containing ZnO-decorated CNT, as shown in Figure 9j, could also exhibit reversible nonlinear conductive behavior, but the filler content of samples is 10% and the nonlinear coefficients only range from four to five [30]. So, the nonlinear conductive characteristics and the fabrication cost of GNPs-CNTs-ZnO/ER composites in this article are obviously better and less than the composites fabricated by Yu et al. respectively. Meanwhile, for possessing similar reversible nonlinear conductive behavior, the need of filler content of GNPs-CNTs-ZnO/ER composites is 2.5%, which is only 12.5% of that in GNPs-ZnO/ER composite reported in our previous work.

In summary, GNPs-CNTs-ZnO/ER composites with appropriate weight ratios of GO to MWCNTs and GNPs-CNTs hybrid to Zn(Ac)_2_ are able to exhibit excellent reversible nonlinear conductive behavior. In addition, their nonlinear coefficients and switching threshold voltages can be controlled by tuning the weight ratios of GO to MWCNTs and GNPs-CNTs hybrid to Zn(Ac)_2_, which is more practical and viable for practical overvoltage protection. When comparing the data in Table 2 and Table 3, it can be observed that adjusting the weight ratios of GNPs-CNTs hybrid to ZnO can result in different advantages in the field of switching threshold voltage, standard deviation, or nonlinear coefficient. Moreover, the group with 1:1 weight ratio of GO to MWCNTs (A-6.67 and A-10) has proper nonlinear coefficients and switching threshold voltage and best reversibility, which could be the most practical GNPs-CNTs-ZnO/ER sample with excellent reversible nonlinear conductive behavior.

**Figure 10:**
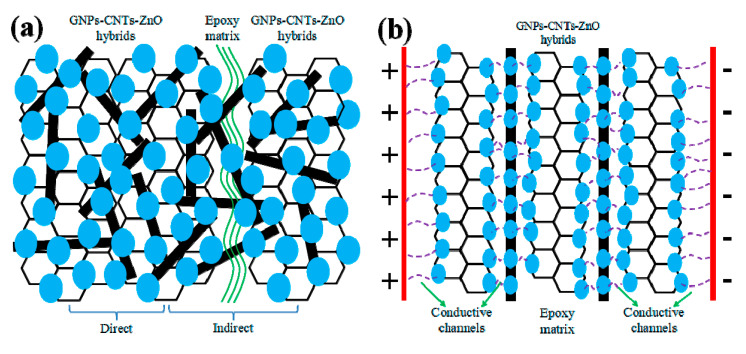
(**a**) Schematic illustration of GNPs-CNTs-ZnO hybrid in the composite and (**b**) the GNPs-CNTs-ZnO/ER structure at high applied voltage sweeps.

To investigate mechanism behind the nonlinear conductive behavior of inhomogeneous materials, researchers have focused on the electronic hopping, field-enhancing tunneling, filler-matrix charge transfer, and filamentary conduction [37,38,39,40,41] As GNPs and CNTs are blended, available contact between GNPs flakes are filled with CNTs. This can substitute most of the insulating thin ER matrix between the GNPs flakes, and thereby effectively improving the conductivity of the hybrid. Meanwhile, an equipotential model is established due to the strong bonds formed evenly between the GNPs-CNTs hybrid and ZnO nanoparticles. This allows the free electrons near the Fermi level of GNPs and CNTs to be effectively transferred to ZnO nanoparticles under an applied voltage. The transferred electron in ZnO nanoparticle can then be further transported to its neighboring ZnO nanoparticles, or it can be transported through the sufficiently thin ER matrix. As such, the conductive paths in GNPs-CNTs-ZnO/ER composite can be considered to be comprised of (1) GNPs-CNTs hybrids-ZnO heterojunctions and (2) ZnO-ER-ZnO. Based on the collective analyses, the mechanism of nonlinear conductive behavior of GNPs-CNTs-ZnO/ER composite is presented in Figure 10.

Figure 10a presents the internal microstructure model of GNPs-CNTs-ZnO hybrid and ER matrix in composites. Due to the excellent conductivity, good internal contact, and extremely high specific surface area, GNPs-CNTs hybrid is the most essential component in the conductive paths. While, the key to the reversibility observed in this work is due to the even distribution of ZnO nanoparticles on the surface of GNPs-CNTs hybrid. As presented in Figure 10a, two different types of connection can be observed between the fillers in composites as described in the following: (1) direct connection whereby ZnO nanoparticles are in direct contact with each other to form GNPs-CNTs hybrid-ZnO-ZnO-GNPs-CNTs hybrid unit. Since the band gap of ZnO nanoparticles is much larger as compared to that of GNPs-CNTs hybrid [42,43] a conventional Schottky barrier is formed between the interface of GNPs-CNTs hybrid and ZnO nanoparticles. This would in turn fulfill the requirement of quantum tunneling effect [44], which allows the electrons to jump from GNPs-CNTs hybrid to ZnO nanoparticles. Due to the hopping and migration of electron at sufficient applied voltage, this would result in the generation of electric current. As such, the GNPs-CNTs hybrid-ZnO-ZnO-GNPs-CNTs hybrid units can be considered as double-Schottky barriers, which can lead to the nonlinear conductive behavior. (2) indirect connection is another type. Though most of fillers are connected by CNTs because of their extremely high aspect ratio, there is inevitably still insulating thin enough ER matrix between GNPs-CNTs-ZnO hybrids and builds the ZnO-epoxy-ZnO units, which is similar with the metal-insulator-metal model with a double-Schottky barrier like direct connection [45,46].

Based on related researches on the double-Schottky barrier [47,48], electrons at high or low energy level of GNPs-CNTs hybrids and ZnO nanoparticles could traverse the potential barrier by hopping effect or quantum tunneling, respectively, which can generate a current in the double-Schottky barrier, according to the equations as shown below.

(1) $$I_{h}=\frac{V}{R_{0}}\exp\left(\frac{eV}{k_{0}T}\right)$$

(2) $$I_{t}=\alpha \left(V+\beta V^{3}\right)$$

(3) $$I=\alpha V+\alpha \beta V^{3}+\frac{1}{R_{0}}V\cdot \exp\left(\frac{eV}{k_{0}T}\right)$$

where both α and β are constants, $I_{h}$ is the generated current due to hopping effect, $I_{t}$ is the generated current due to tunneling effect, and V is the voltage applied. Hence, GNPs-CNTs-ZnO/ER composite behaves like an insulator at relatively low applied voltage. Electron hopping and tunneling effect may happen between the neighboring GNPs-CNTs-ZnO hybrids through the sufficiently thin ER layers as the applied voltage approaches the switching threshold voltage. This could result in GNPs-CNTs-ZnO/ER composite exhibiting nonlinear conductive behavior, with some slight divergence during the initial test. In summary, quantum tunneling and electron hopping at the double-Schottky barrier of GNPs-CNTs hybrid-ZnO-ZnO-GNPs-CNTs hybrid heterojunction and ZnO-epoxy-ZnO unit are the factors that result in the nonlinear conductive behavior exhibited by GNPs-CNTs-ZnO/ER composite.

GNPs-CNTs-ZnO/ER after repeated applied voltage sweeps is illustrated in Figure 10b. Conductive paths can be established between neighboring ZnO nanoparticles with appropriate filler content and weight ratios of GO to MWCNTs and GNPs-CNTs hybrids to Zn(Ac)_2_, due to Joule heating of sufficiently thin insulating ER matrix layer under the sufficiently high applied voltage. This will lead to the formation of a more reversible and stable unit, whereby the conductive paths of electron transport are transformed from ZnO-epoxy-ZnO to GNPs-ZnO-ZnO-GNPs. Furthermore, due to the blending of CNTs with ultra-high aspect ratio, the number of ZnO-epoxy-ZnO units can be effectively decreased in ZnO-coated CNTs. This increases the ease of formation of conductive paths and, simultaneously, improving the stability of the nonlinear conductive behavior. Thus, GNPs-CNTs-ZnO/ER composite is not only able to exhibit excellent nonlinear conductive behavior, it can also demonstrate stable reversibility.

## 4. Conclusions

The reversible nonlinear conductive characteristic of GNPs-CNTs-ZnO/ER composite is elucidated in this work. Based on the result, it is shown that with the with the proper weight ratio of GO to MWCNTs and GNPs-CNTs hybrids to Zn(Ac)_2_, the GNPs-CNTs-ZnO/ER composite is able to exhibit excellent and stable reversible nonlinear conductive behavior with high nonlinear coefficient under a particular applied voltage. In addition, a lower amount of filler is required (only 12.5% of that of GNPs-ZnO/ER composites in our previous research), which can reduce the fabrication cost. These advantages highlight the superiority of the reported GNPs-CNTs-ZnO/ER composite as compared to the previously reported pristine GNPs/ER composites. Meanwhile, the threshold voltage of GNPs-CNTs-ZnO/ER composites could be modulated by adjusting the weight ratio of GO to MWCNTs and GNPs-CNTs hybrids to Zn(Ac)_2_ in the fillers, which provides more parameters for controlling the nonlinear conductive characteristic of the composites. As such, these advantages position the reported GNPs-CNTs-ZnO/ER composite as a more viable material for the practical protection of electronic devices from overvoltage. This work can be an essential addition in the fabrication of graphene polymeric composites for the overvoltage protection application. Moreover, the electron tunneling and hopping due to the GNPs-CNTs hybrid-ZnO-ZnO-GNPs-CNTs hybrid heterojunction and double-Schottky barrier of ZnO-epoxy-ZnO unit both contribute to the reversible nonlinear conductive characteristic exhibited by the GNPs-CNTs-ZnO/ER composite. Finally, the group with the 1:1 weight ratio of GO to MWCNTs (A-6.67 and A-10) exhibits proper nonlinear coefficients and switching threshold voltage, with the best reversibility. This material can be the most practical GNPs-CNTs-ZnO/ER composite with excellent reversible nonlinear conductive behavior.

## Figures and Tables

**Table 1:** Classification and allographs of GNPs-CNTs-ZnO/ER samples.

GNPs-CNTs-ZnO/ER Composites			
Group	Weight Ratio (GNPs to MWCNTs)	Weight Ratio (GNPs-CNTs to Zn(Ac)_2_)	Allographs
A	1:1	1:5	A-5
		1:6.67	A-6.67
		1:10	A-10
		1:20	A-20
B	1:2	1:5	B-5
		1:6.67	B-6.67
		1:10	B-10
		1:20	B-20
C	2:1	1:5	C-5
		1:6.67	C-6.67
		1:10	C-10
		1:20	C-20

**Table 2:** Average nonlinear coefficients values of GNPs-CNTs-ZnO/ER samples of six different samples.

GNPs-CNTs-ZnO/ER Composites		
Sample Allographs	Region 1	Region 2
A-10	1.85	43.80
A-6.67	1.11	42.62
B-10	1.43	31.88
B-6.67	0.58	23.98
C-10	1.34	90.00
C-6.67	1.78	35.16

**Table 3:** Switching threshold voltage of GNPs-CNTs-ZnO/ER samples of six different samples.

GNPs-CNTs-ZnO/ER Composites		
Sample Allographs	Range (V)	Δ (%)
A-10	144.1 ± 3.0	2.08
A-6.67	118.6 ± 4.5	3.79
B-10	115.6 ± 1.5	1.30
B-6.67	67.6 ± 16.5	24.41
C-10	219.2 ± 10.5	4.79
C-6.67	153.2 ± 12.1	7.90
